# Feasibility of AI-driven multichannel FES-assisted gait and cycling training in chronic neurological disorders: a case series

**DOI:** 10.1186/s12984-026-01953-4

**Published:** 2026-03-19

**Authors:** Nikola Babić, Bernardo Fernandes De Sousa, Jonathan Levy, Fleur Touzard, Sophie Blancho, Perrine Séguin, Amine Metani, Maël Descollonges

**Affiliations:** 1 Kurage, Lyon, France; 2 Institut Pour La Recherche Sur La Moelle Épinière (IRME), Station Debout, Paris, France; 3 Service de Médecine Physique Et de Réadaptation, Hôpital Raymond Poincaré (AP-HP Saclay), Garches, France; 4 Laboratoire Sur Les Interactions, Cognition, Action, Émotion (LICAE), Université Paris Nanterre, Garches, France; 5 Laboratoire de Physique, Université de Lyon, École Normale Supérieure de Lyon (ENS Lyon), Centre National de La Recherche Scientifique (CNRS), Garches, France

**Keywords:** Functional electrical stimulation, Gait rehabilitation, Chronic impairments, Spinal cord injury, NeuroSkin®, Feasibility

## Abstract

**Background:**

Gait impairments following chronic neurological conditions, such as spinal cord injury (SCI), represent a major challenge for rehabilitation, with limited options to sustain functional recovery and patient engagement. Functional electrical stimulation (FES) has shown potential to enhance gait performance, but its integration into routine rehabilitation requires clinically feasible and acceptable solutions.

**Objective:**

To assess the feasibility, safety, and acceptability of integrating NeuroSkin®, a wearable AI-powered multichannel FES system, into walking rehabilitation programs for individuals with chronic neurological gait impairments, and to explore its potential to improve functional outcomes.

**Methods:**

This retrospective case series included four individuals (three males, one female; aged 54–78 years) with chronic neurological gait impairments of diverse etiologies (acute disseminated encephalomyelitis, idiopathic transverse myelitis, incomplete spinal cord injury, and polyradiculoneuritis). Participants underwent 20 rehabilitation sessions over 6 weeks, which combined FES-assisted cycling and ambulation with the NeuroSkin device. The system integrates inertial measurement units and plantar pressure sensors to deliver phase-specific stimulation across multiple lower limb muscle groups. Feasibility was assessed through completion rates and adherence to the study protocol. Gait, balance, and spasticity outcomes were recorded before (PRE) and after the intervention (POST) with standardized assessments (10-Meter Walk Test, 2-Minute Walk Test, Berg Balance Scale, Stand Chair Test, Modified Ashworth Scale, SCATS, and Neuropathic Pain Diagnostic Questionnaire).

**Results:**

All participants completed the intervention, demonstrating good feasibility, tolerance, and adherence. Improvements were observed in walking speed and distance (10mWT and 2MWT), with concomitant improvement in lower limb strength and reductions in spasticity for three patients. These findings support the potential integration of AI-driven multichannel FES into long-term gait rehabilitation programs for individuals with chronic neurological conditions.

**Conclusions:**

The integration of the NeuroSkin AI-driven FES system into rehabilitation programs for individuals with chronic neurological gait impairments was feasible, well accepted by patients, and safe. Furthermore, while improvements in gait performance were observed, these findings should be interpreted as exploratory and require confirmation in controlled studies.

## Introduction

Gait impairments are frequent consequences of chronic neurological disorders, including spinal cord injury (SCI), and persistent sequelae of conditions such as Guillain-Barré syndrome, acute disseminated encephalomyelitis (ADEM), or transverse myelitis and constitute a major cause of long-term disability [1, 2]. These conditions often result in paralysis, weakness, or spasticity, markedly restricting independence, mobility, and quality of life. For individuals with neurological gait deficits, restoring walking function is one of the primary health priorities, and further improvement of walking remains a key goal for those with residual walking ability [3–5]. However, despite progress in neurorehabilitation, effective approaches for longstanding deficits remain limited, and sustaining active patient participation and adherence to rehabilitation programs remains challenging.

Functional electrical stimulation (FES) has been widely investigated as a means to enhance motor recovery and facilitate walking in individuals with neurological impairments [6–8]. By activating paralyzed or weak muscles in a task-specific manner, FES can improve gait kinematics, reduce perceived effort, reduce spasticity, and potentially promote neuroplasticity [9–12]. However, the clinical implementation of multichannel FES is often constrained by technical complexity, time-consuming setup, and limited adaptability to individual gait patterns, hindering its routine use in rehabilitation settings.

Recent technological developments have introduced wearable and sensor-integrated systems designed to overcome these limitations [4, 13, 14]. In particular, the NeuroSkin® device (Kurage, France) combines textile-integrated electrodes with inertial measurement units and plantar pressure sensors [13, 15]. By enabling real-time detection of gait phases, the system can deliver synchronized, phase-specific stimulation across multiple lower-limb muscle groups. The machine learning algorithms embedded in the FES-NeuroSkin® system analyze real-time data from the inertial measurement units (IMU) and plantar pressure sensors to identify gait phases [13, 15]. Based on these inputs, the algorithms automatically adjust the timing and sequencing of electrical stimulation for multiple lower-limb muscles to optimize gait symmetry, coordination, and functional efficiency [13].

Although prior studies from our group have demonstrated the feasibility of the NeuroSkin® system in subacute stroke patients [13], gait impairment patterns vary substantially across neurological conditions, including spinal cord injury and chronic transverse myelitis. The NeuroSkin® system is designed to be adaptable across different neurological populations, as stimulation parameters can be individually calibrated and modulated according to residual motor capacity, spasticity, and patient comfort. However, there is a lack of literature about the feasibility and effectiveness of this AI-driven, real-time gait-phase classification and adaptive functional electrical stimulation in chronic neurological populations beyond stroke. Moreover, the impact on chronic neurological impairment must be explored. This uncertainty further supports the need to investigate the feasibility, safety, and potential preliminary functional benefits of AI-driven multichannel FES-assisted gait training in a broader range of chronic neurological gait disorders.

Furthermore, evidence supporting the use of AI-driven multichannel FES in patients with neurological impairments, including SCI, Guillain-Barré syndrome, ADEM, and transverse myelitis, remains scarce. To date, feasibility studies conducted in post-stroke patients and incomplete SCI reported promising results, suggesting that multichannel FES-assisted gait training is both safe and potentially beneficial [4, 13]. Case series studies can provide valuable preliminary insights into the feasibility, safety, and potential benefits of such systems, and can help design larger clinical trials. Thus, the present study aimed to assess the feasibility, adherence, and safety of integrating the NeuroSkin® multichannel FES system into walking and cycling rehabilitation programs for individuals with chronic neurological gait impairments. A secondary objective was to explore its potential impact on functional outcomes, including gait performance, muscle tone (spasticity), neuropathic pain, and motor function.

## Materials and methods

### AI-powered FES system

**Fig. 1:**
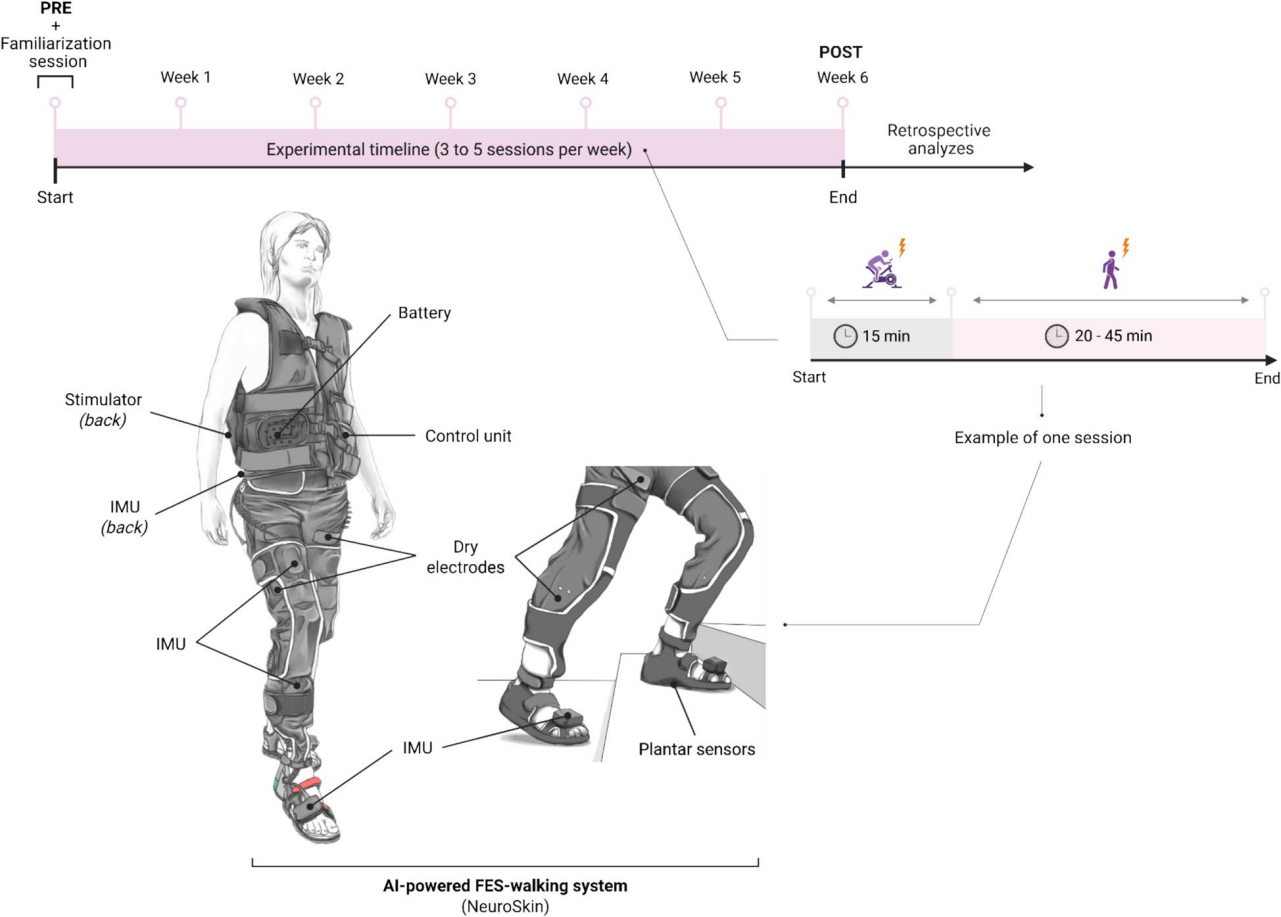
Illustration of the experimental timeline and an AI-driven multichannel FES-assisted walking session with the NeuroSkin® system. The participant wears a NeuroSkin device equipped with textile electrodes and embedded sensors (inertial and plantar pressure sensors). A physiotherapist supervises the session and adjusts stimulation parameters in real-time through the NeuroSkin® tablet interface and a remote control

The NeuroSkin device (NeuroSkin, Kurage, France), a CE-marked Class IIa medical device, consists of tight-fitting pants worn by participants during walking. It functions as both a therapeutic tool and a real-time gait monitoring platform, designed to enhance walking function by delivering electrical stimulation to the lower limbs at precisely timed intervals [13, 15] (Fig. 1).

The device includes a lower-extremity garment with embedded FES dry electrodes targeting six muscle groups bilaterally: Gluteus Maximus, Quadriceps, Hamstrings, Tibialis Anterior, Fibularis Longus, and Gastrocnemius. Muscle activation was synchronized with specific gait phases: the Gluteus Maximus and Quadriceps were stimulated during the stance phase to support weight bearing, while the Hamstrings, Tibialis Anterior, Fibularis Longus, and Gastrocnemius were stimulated during swing and push-off phases to facilitate foot clearance, propulsion, and overall gait coordination.

It also incorporates seven inertial measurement units (IMUs) positioned on the pelvis, upper and lower leg segments, and feet, and eight ground reaction force (GRF) sensors embedded in the shoe insoles. In this version, the IMUs are embedded in an additional textile layer attached to the pants using hook-and-loop fasteners rather than being integrated directly into the garment [12, 13]. A microcomputer mounted on the back of the vest worn by the participant hosts an AI-based real-time gait phase detector. The system further includes a MotiMove electrical stimulator (3F-Fit Fabricando Faber, Belgrade, Serbia) placed in the back of the device, a remote controller for adjusting stimulation intensity, and a software application that allows therapists to configure individual patient profiles and stimulation parameters [12, 13].

Stimulation was delivered at a frequency of 40 Hz for all targeted muscle groups. Pulse widths were set to 400 µs for the Quadriceps, Gluteals, and Hamstrings; 250 µs for the Triceps Surae; and 200 µs for the Tibialis Anterior and Fibularis Longus muscles. These parameters were selected based on the device’s recommendations, the physiotherapist's clinical expertise, established FES guidelines [16], and FES parameters previously used in studies from our research group [11, 17, 18]. At the start of each session, stimulation intensity was set to the participant’s maximum tolerable level, as determined by their subjective perception, to optimize therapeutic effect and minimize functional deficits. No formal recruitment curve was obtained; the intensity was gradually increased by the therapist until the participant reported reaching their comfort limit. Adjustments could be made in real time during the session to accommodate fatigue or changes in sensation. Therapists could make real-time adjustments via the NeuroSkin® interface to accommodate individual gait deficits and maintain comfort. Consistent with prior recommendations [19], a small amount of moisturizing lotion (CeraVe Moisturizing Lotion, L’Oréal USA, New York) was applied to the skin at electrode sites before donning the pants to ensure adequate epidermal hydration and improve electrode–skin adhesion.

As described previously by our group [13], the NeuroSkin® hardware is operated via a tablet application, which includes two primary interfaces: the Global Settings screen and the Expert Settings screen [13]. The Global Settings screen enables the operator to manage multiple patient profiles, including demographic information, paretic side, and maximum permitted stimulation intensities for each muscle group. It also allows monitoring of hardware connections and controls for session start, pause, and stop. The Expert Settings screen provides access to advanced parameters, including the global stimulation frequency for all channels, as well as channel-specific pulse widths and timing expressed as a percentage of the gait cycle. Data from the embedded sensors are processed in real time to determine gait phase percentages, which are then used to trigger electrical stimulation according to predefined rules tailored to the patient’s individual gait pattern [20]. The same mechanism also allows FES-assisted cycling, stair ascent, and stair descent.

Before the first session, each participant undergoes calibration and personalization procedures. Calibration involves identifying the maximum stimulation intensity that the participant can comfortably tolerate for each targeted muscle. The system is then programmed to limit stimulation to these maximum values. These limits can be adjusted during subsequent sessions as needed, for instance, to account for habituation or fatigue. The inertial measurement units (IMUs) used for gait phase detection are initially factory-calibrated and do not require manual recalibration before each session. Their functional adaptation to each participant is performed automatically during the personalization procedure described below.

Both personalization and calibration are only required before the initial session and typically do not need to be repeated during the intervention. However, they can be performed again if the therapist considers it necessary due to significant improvements in the patient’s walking ability. Personalization is performed by having the patient walk approximately 20 steps without electrical stimulation. The gait data collected during this trial are used to partially retrain the general gait model, originally developed using deep learning on a dataset of 32 healthy and post-stroke hemiplegic walking patterns, so that it better reflects the individual’s specific gait characteristics [13]. This refinement improves the accuracy of real-time gait phase classification and, consequently, the timing of stimulation delivery [13].

### Gait rehabilitation sessions

Sessions were conducted in a clinical research center. One single physiotherapist supervised all sessions with AI-powered FES, always positioned near the patient to ensure safety and proper execution of the session (Fig. 1).

Each session began with a 15-min FES-assisted cycling warm-up using either the Motomed Viva 2 Light or Motomed Muvi (RECK-Medizintechnik GmbH & Co. KG, Betzenweiler, Germany), performed in “assistance mode” while wearing the NeuroSkin®. Cadence was primarily self-selected by the participants and adjusted when necessary, by the therapist according to each participant’s residual motor capacity, fatigue level, and spasticity. On average, cadence ranged between 30 and 50 rpm. This individualized approach aimed to maintain smooth and controlled leg movements, minimize the risk of spasticity exacerbation, and allow safe habituation to electrical stimulation before walking exercises. Beyond its neuromuscular warm-up role, FES-assisted cycling was selected as a preparatory task due to its documented benefits on exercise tolerance and central hemodynamic responses in individuals with stroke [11]. Participants then completed a FES-assisted walking session at a self-selected comfortable pace, either in flat indoor corridors or in outdoor environments. If required, walking sessions were carried out with technical aids (e.g., canes or a walking frame). Walking sessions included repeated overground walking, with stair ascent and descent in three of the four patients, based on their functional capacities. The decision for a participant to attempt specific gait tasks was primarily guided by the physiotherapist’s evaluation of residual motor function, balance, and safety, while taking participant preference into account whenever feasible. Sessions typically alternate between short walking bouts and brief rest periods, depending on patient fatigue and tolerance. Walking time in each session lasted between 20 and 45 min. The duration of each session was individualized rather than fixed, and the physiotherapist could pause or terminate the session if signs of muscle fatigue, discomfort, or spasticity were observed. This flexible approach allowed participants to train effectively while minimizing the risk of overfatigue. Each participant completed 20 sessions over 6 weeks, with a frequency of three to five sessions per week. In line with usual clinical rehabilitation practice, sessions were generally constrained to approximately one hour of therapeutic activity, and no session exceeded this duration.

### Participant’s experience with FES—qualitative data

Following the FES rehabilitation program, the physiotherapist informed each patient that they were free to share their experience with the FES-assisted cycling and walking training if they wished to do so. No predefined questions or interview guide were used. Only participant 1 agreed voluntarily to provide feedback regarding satisfaction (see the results part). With the patient’s explicit consent, comments were transcribed verbatim on paper in an anonymous manner.

### Gait parameters

As previously presented, a secondary objective was to explore the potential impact of the NeuroSkin’s FES system on functional outcomes, including gait performance, spasticity, and motor function.

The 2-Minute Walk Test (2-MWT) was used to evaluate walking speed, walking capacity, and endurance [21]. The 2-Minute Walk Test (2MWT) was conducted on a 10-m pathway marked by two cones. Participants were instructed to walk straight for 10 m, around the cones, and back, with or without assistive devices, for two minutes to cover the greatest possible distance. They were allowed to slow down or stop if necessary, and the total distance walked (in meters) was recorded. In addition, the 10-Meter Walk Test (10-mWT) was conducted in self-selected speed to assess walking speed. For this test, participants walked a total distance of 10 m, with or without the use of assistive devices.

Furthermore, walking speed was derived from the inertial measurement units (IMUs) integrated into the NeuroSkin® device. One IMU was positioned on the dorsal side of each foot (right and left), allowing real-time estimation of gait velocity. This continuous measurement provided additional robustness to our assessment of walking performance, which complemented the conventional 10-mWT and 2-MWT. Considering the inter-session variability (related to fatigue, motivation, or daily condition), we compared the mean walking speed from the first five sessions with that from the last five sessions for each participant.

### Balance parameter

Participants’ balance performance was assessed using the Berg Balance Scale (BBS), a clinical tool commonly used in neurological rehabilitation [22], to evaluate postural control and fall risk. The scale comprises 14 items, each scored 0 to 4, yielding a maximum score of 56, with higher scores reflecting better balance ability [23].

### Lower limb muscle strength

We also used the 30-s Chair Stand Test to assess lower limb strength [24]. Participants were asked to stand up fully from a seated position in a standard chair and then sit back down, repeating this as many times as possible within 30 s.

### Muscle tone parameter

Muscle tone was assessed using the Modified Ashworth Scale (MAS) [25] and with the Spinal Cord Assessment Tool for Spastic Reflexes (SCATS) [26] for participants 1, 2, and 3.

### Neuropathic pain

Neuropathic pain was evaluated for participants 1 and 3 using the DN4 Neuropathic Pain Diagnostic Questionnaire [27]. Participants 2 and 4 did not have any neuropathic pain. These evaluations were also performed at PRE and POST intervention.

### Data collection

This retrospective observational study was conducted in Station Debout Research Center in accordance with the latest version of the Declaration of Helsinki. The data were collected in the first 2025’s semester and processed in compliance with the General Data Protection Regulation (GDPR), ensuring the protection of individuals' privacy and personal data. Authorization to use the data was obtained from the National Commission for Data Protection and Privacy (CNIL: 2,227,969 v 0, MR-004). Ethics compliance, data collection, and analysis were carried out in accordance with the MR004 reference methodology. Participants were informed that data obtained from the NeuroSkin® system and from the standardized assessments would be used for research purposes, and that they could object to it at any time.

## Results

### Participants characteristics

Participants were selected through a qualitative assessment among individuals who had previously used the device at the research center and expressed interest in joining a longitudinal rehabilitation program. Eligible participants presented with a chronic neurological disorder of central or peripheral origin and a minimal to moderate locomotor capacity. Following application of the inclusion and exclusion criteria, four participants (three males, one female; aged 54–78 years) with chronic neurological gait impairments of various etiologies were included in this retrospective case study. All participants had long-standing, medically stable conditions with sufficient residual voluntary motor control for assisted ambulation and no contraindications to FES-based training. Additional eligibility criteria included muscle tone ≤ grade 3 on the Modified Ashworth Scale, preserved sensation, and adequate cognitive and motivational capacity. Exclusion criteria encompassed the presence of implantable electrical devices, uncontrolled hypertension, and significant lower-limb contractures.

Participant 1 (P1) was a 54-year-old woman (50 kg, 169 cm) diagnosed with acute disseminated encephalomyelitis (ADEM) 19 years before enrollment. At the time of inclusion, neurological impairment was at the T12 level, with an admission score corresponding to American Spinal Injury Association Impairment Scale (AIS) grade D. She demonstrated the ability to ambulate short distances with bilateral canes.

Participant 2 (P2) was a 55-year-old man (95 kg, 177 cm) with idiopathic transverse myelitis, diagnosed 9 years before enrollment. His neurological level of injury was identified at T8, and he presented with an AIS grade C classification. He was capable of ambulation using a walking frame or bilateral forearm crutches, during which compensatory trunk movements, including forward trunk flexion and lateral trunk shifts, were observed to assist with balance and weight transfer.

Participant 3 (P3) was a 72-year-old man with a history of incomplete traumatic spinal cord injury sustained in a motor vehicle accident 5 years prior. The injury was localized at the C7 level, and the participant was classified as AIS grade D. He demonstrated functional ambulation using bilateral canes for support.

Participant 4 (P4) was a 78-year-old man (80 kg, 188 cm) with a history of polyradiculoneuritis (Guillain-Barré syndrome), with symptom onset 15 years before participation in the program. He exhibited flaccid weakness of the lower limbs, consistent with peripheral motor involvement, and ambulated with the assistance of two walking poles. Participants’ demographic and clinical characteristics are available in Table 1.

**Table 1:** Baseline demographic and clinical characteristics of the 4 participants

	Participant 1	Participant 2	Participant 3	Participant 4
Age (years)	54	55	72	78
Gender	F	M	M	M
Weight (kg)	50	95	63	80
Height (cm)	169	177	174	188
Diagnosis	ADEM	Idiopathic myelitis	Incomplete SCI	Polyradiculoneuritis
Year of onset	2006	2016	2020	2010
Level of the lesion	T12	T8	C7	–
Admission score	AIS D	AIS C	AIS D	–
Walking aid	Two canes	Walking frame	Two canes	Two walking poles

ADEM: Acute Disseminated Encephalomyelitis; SCI: Spinal Cord Injury; AIS: American Spinal Cord Injury Association Impairment Scale grade; F: Female; M: Male

### FES-assisted walking session duration

Means of FES-assisted walking session duration per participant were: P1: 23 ± 10; P2: 31 ± 7; P3: 24 ± 12; P4: 26 ± 9 min).

### Stimulation parameters

Pulse widths were set to 400 µs for the quadriceps, gluteals, and hamstrings, 250 µs for the gastrocnemius, and 200 µs for the tibialis anterior and fibularis longus muscles. Mean stimulation intensities applied to each muscle group varied across participants. Electrical stimulation amplitudes (mA) ranged from 33.5 to 44 mA for the quadriceps, tibialis anterior from 25.5 to 46 mA, fibularis longus from 25.5 to 44.5 mA, hamstrings from 31.5 to 51 mA, gluteal muscles from 37.5 to 44.5 mA, and gastrocnemius from 17.5 to 69 mA. Stimulation intensities (mA) applied for each participant, and each stimulated muscle are available in Table 2.

**Table 2:** Total number of sessions performed, mean (± SD) FES-assisted walking session duration (minutes), and mean (± SD) stimulation intensities (mA) applied for each participant and each stimulated muscle

	Participant 1	Participant 2	Participant 3	Participant 4
Number of sessions	20	20	20	20
Time of walking sessions (minutes)	23 ± 10	31 ± 7	24 ± 12	26 ± 9
Quadriceps intensity (mA)	44 ± 20	33.5 ± 2	34.5 ± 6	43 ± 3
Hamstrings intensity (mA)	32.5 ± 5	33.5 ± 1	31.5 ± 1	51 ± 4
Gluteus intensity (mA)	39 ± 3	41.5 ± 1	37.5 ± 8	44.5 ± 1
Gastrocnemius intensity (mA)	20 ± 3	17.5 ± 1	33.5 ± 5	69 ± 1
Tibialis Anterior intensity (mA)	25.5 ± 2	46 ± 3	38.5 ± 1	39 ± 3
Fibularis Longus intensity (mA)	25.5 ± 2	26 ± 1	39.5 ± 2	44.5 ± 8

### Feasibility, safety, and adherence

The multichannel FES-assisted gait training was well tolerated and positively received by participants, as reflected by their full adherence to the program and favorable verbal feedback. Participant 1 reported an unpleasant sensation in one gluteal muscle; stimulation of that muscle was discontinued for the remainder of the session, and the issue was resolved with a size adjustment. Moreover, one adverse event was reported for participant 2: bilateral, reversible leg swelling at the end of the program. This illustrates the importance of personalized fitting for comfort and safety. Aside from this, no other adverse events or significant discomfort related to either the stimulation or the device were observed. Notably, all participants completed the 6-week intervention, achieving a 100% session completion rate.

### Participant’s experience with FES—qualitative data

Participant 1 reported a generally positive experience with the FES-assisted walking training, stating: *“Since I’ve been using the system regularly, I’ve noticed a clear improvement in the knee recurvatum in both sides.”* She also said: *“When I use the system, I no longer have to focus on everything I need to do to walk, like bending the knee or lifting the hip.”* According to the participant, *“it becomes automatic, so I can concentrate on my balance instead,”* and *“you can have a conversation, think about something else, it is really pleasant.”*

### Gait and balance parameters

**Fig. 2:**
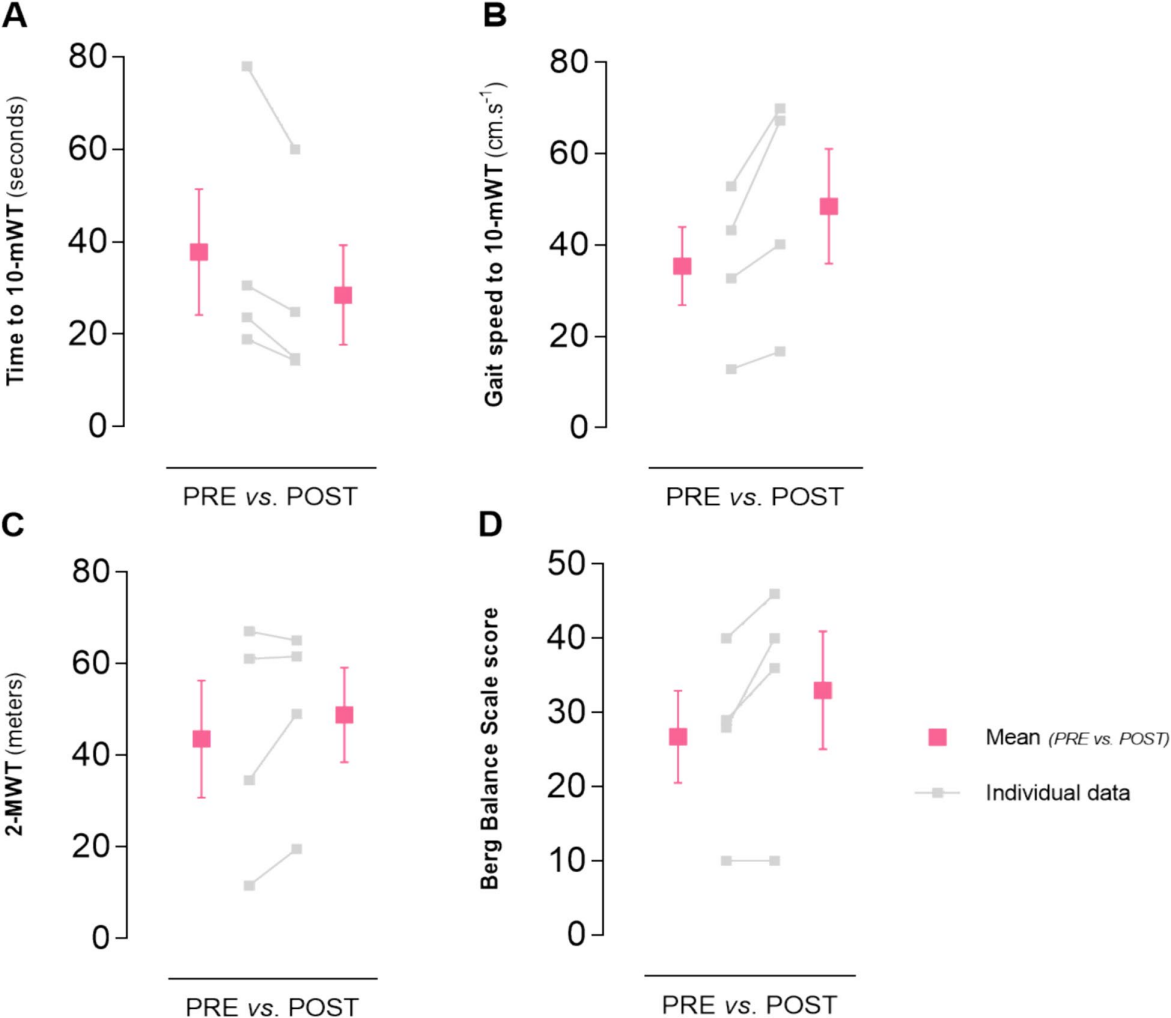
Walking time (**A**) and gait speed during the 10-mWT (**B**), 2-MWT performance (**C**), and Berg Balance Scale score (**D**). Data are presented as mean (purple squares) ± SD, along with individual values for each participant at PRE and POST intervention. The figure was created via GraphPad Prism

As shown in Fig. 2 and Table 3, both walking time (Panel A; Participant 1: − 18.7%, Participant 2: − 23.1%, Participant 3: − 37.2%, Participant 4: − 24.4%) and gait speed during the 10-mWT (Panel B; Participant 1: + 23%, Participant 2: + 30%, Participant 3: + 55.4%, Participant 4: + 32.2%) showed consistent improvements across all participants from pre- to post-intervention. Three out of four participants exceeded the established minimal clinically important difference (MCID) for the 10-mWT in individuals with SCI (0.06–0.13 m·s⁻^1^) [28, 29], with a mean improvement of 0.13 m·s⁻^1^ (P1: + 0.075; P2: + 0.039; P3: + 0.240; P4: + 0.170 m·s⁻^1^, respectively), indicating clinically meaningful gains in walking speed.

**Table 3:** Individual values and means changes in walking performance and balance outcomes following the training intervention

Participants	10-mWT *(seconds)*		10-mWT *(cm.s*^*−1*^*)*		2-MWT *(meters)*		Berg Balance Score		Mean walking speed *(cm.s*^*−1*^)*	
	PRE	POST	PRE	POST	PRE	POST	PRE	POST	PRE	POST
1	30.58	24.87	32.7	40.21	34.5	49	29	36	14.4	17.6
2	78	60	12.82	16.67	11.5	19.5	10	10	8.9	11.8
3	23.68	14.88	43.23	67.2	61	61.5	40	46	27.3	26
4	18.92	14.31	52.87	69.89	67	65	28	40	37.1	45.4
Mean change	− 25%		+ 37%		+ 12%		+ 23%		+ 14%	

The table presents pre- and post-intervention values for the 10-m walk test (10-mWT; walking time and gait speed), the 2-min walk test (2-MWT; walking distance), the Berg Balance Scale score, and the mean walking speed. Negative values for walking time indicate improvement (i.e., reduced completion time), whereas positive values for gait speed, walking distance, and balance score reflect performance gains. *: data from IMU sensors

Walking distance during the 2-MWT also increased for three participants (Panel C; Participant 1: + 42%, Participant 2: + 69.6%, Participant 3: + 1%), whereas Participant 4 exhibited a slight decrease (− 3%). Finally, the Berg Balance Scale score improved in three participants (Panel D; Participant 1: + 24.1%, Participant 3: + 15%, Participant 4: + 42.9%), while no change was observed for Participant 2.

Mean walking speed (from IMUs) showed heterogeneous changes between the first and last five sessions. Specifically, walking speed increased by 14.3% (P1), 32.6% (P2), and 22.4% (P4), while it decreased by 5.0% (P3).

Overall, these results indicate an improvement in walking performance for three out of four participants, consistent with the positive trends also observed in the 10-mWT and the 2-MWT.

### Lower limb muscle strength

Lower limb muscle strength increased in three of the four participants (+ 18%), as reflected by the greater number of repetitions performed by participants 2, 3, and 4 (+ 4, + 1, and + 1 repetitions, respectively). In contrast, participant 1 was unable to complete the test following the training program.

### Muscle tone parameters

In the three first patients presenting spasticity, a reduction was generally observed in both SCATS (Left/Right vs. PRE; Participant 1: − 100%, Participant 2: − 50/-75%, Participant 3: 0%) and MAS scores (Left/Right vs. PRE; Participant 1: − 67/ − 80%, Participant 2: − 33%/ − 40%, Participant 3: − 62.5%/ − 16.7%). While Participants 1 and 2 showed improvements in both measures, Participant 3 exhibited a decrease in muscle tone on MAS but no change on SCATS. For Participant 4, no modification in muscle tone was detected (Table 4).

**Table 4:** Individual values and means changes in spasticity outcome and neuropathic pain

Participants	MAS *(Left)*		MAS *(Right)*		SCATS *(Left)*		SCATS *(Right)*		DN4	
	PRE	POST	PRE	POST	PRE	POST	PRE	POST	PRE	POST
1	1	0.33	0.55	0.11	2	0	1	0	8	6
2	0.66	0.44	0.55	0.33	4	2	4	1	–	–
3	0.88	0.33	0.66	0.55	0	0	1	1	7	5
4	–	–	–	–	–	–	–	–	–	–
Mean change	− 57%		− 44%		− 67%		− 67%		− 27%	

The table presents pre- and post-intervention values for the MAS, SCATS, and DN4 questionnaire. Negative values for all outcomes indicate improvement (i.e., reduced spasticity level)

### Neuropathic pain

Regarding neuropathic pain assessed using the DN4 questionnaire, both participants showed a reduction in scores following the intervention. Participant 1 demonstrated a decrease from 25% (PRE: 8; POST: 6), while Participant 3 showed a reduction from 28.6% (PRE: 7; POST: 5). These changes indicate a potential alleviation of neuropathic pain symptoms after the intervention.

## Discussion

This preliminary case report aimed to assess the feasibility of the FES NeuroSkin system and investigate its potential to enhance functional outcomes following 20 sessions of multichannel FES-assisted cycling and gait training in four individuals with chronic neurological gait impairments.

Firstly, our results show that this 20-session protocol is both feasible and acceptable. The assessment of feasibility and safety was based on session completion rates, participant feedback, and the incidence of adverse events. All participants completed the 6‑week intervention, with a session completion rate of 100%, which is comparable to the results reported in other feasibility studies in individuals with chronic SCI or neurological disorders [4, 13]. Participants reported positive feedback regarding comfort, usability, and perceived usefulness of the NeuroSkin® system, suggesting high acceptability. Future studies should further quantify user experience using standardized patient-reported outcome measures. Development of home-based AI-assisted FES programs may also help improve accessibility and continuity of rehabilitation.

Objective measures of walking performance revealed improvements following the intervention. Both walking time and gait speed during the 10-mWT showed consistent improvement across all participants, indicating a positive impact on walking efficiency. These findings are consistent with previous studies showing that functional electrical stimulation (FES)-assisted gait training can improve locomotor performance in neurological populations, including patients with spinal cord injury (SCI). For example, studies demonstrated that FES-assisted gait training improved walking speed and endurance in individuals with chronic incomplete SCI [4, 30, 31] or in post-stroke patients [13].

The magnitude of walking speed improvement approached or exceeded thresholds commonly reported as clinically meaningful in some neurological populations, such as in individuals with chronic spinal cord injury (SCI). Improvements of approximately 0.10 m/s in the 10-m walk test have been proposed as representing a minimal clinically important difference (MCID) in SCI populations [28, 29]. In the present case series, three participants demonstrated gains within or above this range. However, no established MCID values are currently available for walking speed in individuals with Guillain-Barré syndrome, ADEM, or transverse myelitis. Therefore, the SCI-derived minimal clinically important difference (MCID) should not be considered directly transferable to these neurological populations but rather used as a comparative benchmark to contextualize the magnitude of observed changes. Given the small and heterogeneous sample and absence of statistical testing, these results should be interpreted cautiously, although observed changes exceeded typical measurement variability and may reflect meaningful functional improvements.

Furthermore, performance during the 2-MWT also improved, suggesting improvements in endurance and functional walking capacity, even many years after disease onset or injury. This is consistent with findings in individuals with incomplete SCI, where FES-assisted walking training led to significant gains in walking distance [32]. In the present study, individual responses were heterogeneous, with changes ranging from + 14.5 m to − 2 m, and a mean improvement of approximately 5 m. No established MCID exists for the 2-min walk test in the neurological populations evaluated in this study. Therefore, changes in 2-MWT performance should be interpreted with respect to measurement properties, such as measurement error and detectable change thresholds, rather than clinical significance thresholds. In walking endurance outcomes, changes of approximately 15 to 30 m have been reported as potentially reflecting clinically meaningful changes in functional walking performance in some neurological rehabilitation populations, particularly for tests such as the 6-Minute Walk Test, although these values should be considered descriptive comparative references rather than validated MCID thresholds for the populations studied [33]. In this case series, one participant demonstrated an improvement above the lower range of reference values, while the remaining participants showed smaller improvements, maintenance of performance, or a slight decline. As mentioned previously, given the small sample size, clinical heterogeneity of participants, and absence of statistical testing, these findings should also be interpreted cautiously.

Berg Balance Scale scores also increased, suggesting beneficial effects not only on locomotor function but also on postural stability. This finding aligns with recent evidence showing that FES interventions targeting proximal lower limb muscles can influence balance control by improving coordination in diverse neurological populations [34]. However, in the absence of established MCID values for balance outcomes in the studied populations, these changes should be interpreted cautiously. Overall, these results suggest potential multidimensional benefits of multichannel FES-assisted training across locomotor and postural domains.

In participants presenting spasticity, decreases were observed in SCATS and MAS scores, indicating a reduction in hypertonia. This finding is consistent with previous findings suggesting that repeated FES training, such as FES-assisted cycling, may contribute to neuromodulatory effects and spasticity management [35]. However, in our study, one participant did not exhibit changes in SCATS (participant 3), which could be related to interindividual variability in responsiveness or limitations of the MAS in detecting subtle modifications in tone. Nevertheless, overall trends support potential benefits of FES for spasticity management in addition to locomotor function.

Taken together, these findings suggest that multichannel FES-assisted cycling and gait training using wearable AI-assisted technology may represent a promising therapeutic approach for chronic neurological disorders, a population in which spontaneous neurological recovery is generally limited after the subacute phase. By simultaneously enhancing walking speed, endurance, balance, and reducing spasticity, this approach may help extend functional independence and quality of life.

However, because the intervention combined FES-assisted cycling during warm-up and multichannel FES-assisted walking training, the present study does not allow disentangling the specific contribution of each intervention component (cycling, walking, or FES) to the observed improvements in gait performance and endurance. The reported effects should therefore be interpreted as the result of the integrated rehabilitation program rather than attributed to a single modality. Nevertheless, this multimodal approach reflects current clinical practice, where complementary task-oriented interventions are commonly combined. The overall impact of the program is reflected in the functional improvements observed in individuals with chronic neurological disorders, a population in whom major spontaneous neurological recovery predominantly occurs within the first months following injury, with limited additional improvement typically expected beyond the subacute phase [2, 36].

There are several limitations to consider when evaluating the results of this study. Firstly, the small sample size and absence of a control group limit the generalizability of these findings. However, the heterogeneity of the sample should not be viewed solely as a limitation. Rather, it was intentionally adopted to explore the adaptability and feasibility of the system across different chronic neurological profiles, rather than to demonstrate pathology-specific efficacy. This methodological positioning aligns with the exploratory and translational nature of the study. Furthermore, the follow-up period was not sufficient to determine whether the observed benefits were sustained over time. Lastly, it is also important to highlight the need for independent validation, meaning confirmation of the results by third parties outside company-sponsored settings. In addition, the combined nature of the intervention does not allow isolation of the specific effects of walking, cycling, or FES, which should be addressed in future studies using factorial or controlled designs. Future studies should therefore include larger cohorts, randomized designs, and long-term follow-up assessments to confirm the robustness and durability of the effects.

In conclusion, this preliminary case report demonstrates that a 20-session program of multichannel FES-assisted cycling and gait training with NeuroSkin is feasible, safe, and well-tolerated in individuals with chronic neurological gait impairments. Improvements in walking speed, endurance, balance, and spasticity suggest that this intervention has the potential to address multiple dimensions of gait impairment and functional mobility. These findings support the potential therapeutic value of integrating advanced wearable FES technologies, such as NeuroSkin, into rehabilitation programs, even in the chronic stages of neurological injury. While promising, these results require confirmation through larger, controlled studies with extended follow-up to establish the long-term efficacy and clinical applicability of this approach.

## Data Availability

De-identified individual participant data (including data dictionary), statistical code, and other related materials are available from the corresponding author upon reasonable request.
